# Metal Nanoparticles for Improving Bactericide Functionality of Usual Fibers

**DOI:** 10.3390/nano10091724

**Published:** 2020-08-31

**Authors:** George Frolov, Ilya Lyagin, Olga Senko, Nikolay Stepanov, Ivan Pogorelsky, Elena Efremenko

**Affiliations:** 1 National Research Technological University “MISIS”, Leninsky ave. 4, 119049 Moscow, Russia; georgifroloff@yandex.ru; 2 Faculty of Chemistry, Lomonosov Moscow State University, Lenin Hills 1/3, 119991 Moscow, Russia; lyagin@mail.ru (I.L.); senkoov@gmail.com (O.S.); na.stepanov@gmail.com (N.S.); 3 N.M. Emanuel Institute of Biochemical Physics RAS, Kosygin str. 4, 119334 Moscow, Russia; 4 48 Central Scientific Research Institute of the Ministry of Defense of the Russian Federation, Oktyabrsky ave. 121, 610017 Kirov, Russia; ipogorelsky@inbox.ru

**Keywords:** metal nanoparticles, fiber material, antibacterial activity, bioluminescent cells

## Abstract

A wide variety of microbiological hazards stimulates a constant development of new protective materials against them. For that, the application of some nanomaterials seems to be very promising. Modification of usual fibers with different metal nanoparticles was successfully illustrated in the work. Tantal nanoparticles have shown the highest antibacterial potency within fibrous materials against both gram-positive (*Bacillus subtilis*) and gram-negative (*Escherichia coli*) bacteria. Besides, the effect of tantal nanoparticles towards luminescent *Photobacterium phosphoreum* cells estimating the general sample ecotoxicity was issued for the first time.

## 1. Introduction

Multiple metal-containing nanomaterials are of great interest since they possess explicit antimicrobial action and could possibly result in absence of microbial resistance to them [1]. This antimicrobial activity (e.g., bacteriostatic and bactericide) of nanomaterials was established to be predetermined by their chemical composition, particle size, shape, morphology, etc. [2]. Further, such antimicrobial functionality can be utilized to produce novel special materials for the food and textile industries, medicine, etc. [3,4].

Various fibrous materials are used in the textile industry, and thus, efficiency of their modification by different nanomaterials can vary significantly. Even micro- [5] and nanofibers [6] were suggested as carriers, though such novel materials certainly can be implemented in a distant future. The fibers currently used in protective outfits are much more common and produced on the basis of synthetic, semi-synthetic, natural polymers, and combinations thereof. Notably, the paradigm of multipurpose functionalization of textiles [7] could be applied for antimicrobial activity also [8].

It should be noted that antimicrobial activity of functionalized materials are usually determined with suspension or solid culture assay. However, both assays appeared to be poorly suited for such materials since: (i) either bacteriostatic or bactericide effect is caused by diffusion of active compound(s) from materials into medium (i.e., ability to diffuse is mostly measured); (ii) cell viability was not issued within materials (i.e., no data about real life application of protective outfits are provided). To solve the issue, specific dyes [9] or even a panel of direct detection bioassays [10] could be adapted. Alternatively, the concentration of intracellular adenosine triphosphate (ATP) [11,12] could be measured to produce highly reproducible and relevant results with nanomaterials [13].

Thus, the purpose of this work was the development of novel fibrous materials functionalized by metal nanoparticles which have antibacterial activity towards both gram-negative and gram-positive bacteria. For that, nanoparticles of various metals (Zn, Fe, Ti, and Ta) were produced, characterized, and deposited onto fibrous materials under varying conditions. Textiles with stable antibacterial activity within the bulk matrix were of special interest.

## 2. Materials and Methods

### 2.1. Preparation of Metal Nanoparticles

Metal nanoparticles were obtained using a unique laboratory device in a plasma electric arc discharge [14]. The main elements of the installation were a power supply unit, a capacitor unit, an air discharger, an electrode unit, a resistance unit, a pressure pump, and a control unit. The installation has the following variable parameters: 5–7 kV voltage supplied to metal electrodes; up to 3000 A current strength (the amplitude of the first half-wave); up to 15 μs electric pulse duration; ca. 100 V residual voltage across the capacitor unit; 0.2–0.3 ms capacitor recharging time; 300–500 Hz frequency of working discharges; 60 rpm rotation rate of the movable electrode relative to the stationary one; up to 10 L/h peristaltic pump performance; 1 bar peristaltic pump pressure; 100–800 μm spark length; and 800–10,200 pF capacitance of the capacitor unit. Specifically, metal nanoparticles were synthesized using a capacitor of 2200 pF, and the air gap in discharger was 500–550 μm (in the case of organic solvents) and 600–650 μm (in the case of distilled water) at ca. 12,000–15,000 K.

Different discharge modes allow the control of the composition and dispersion of nanoparticles. The concentration of nanoparticles was regulated by the performance of the peristaltic pump and by the multiplicity of treatment of the same solution. A single discharge treatment has resulted in metal concentrations of up to 40 μg/mL. The elemental composition of nanoparticles can be controlled by the chemical composition of the electrodes (including multiphase systems) and by the corresponding dispersion medium.

### 2.2. Characterization of Metal Nanoparticles

To determine the size of metal nanoparticles, a transmission electron microscope (TEM), LEO 912 AB OMEGA (Carl Zeiss, Oberkochen, Germany), with energy filter and Keller system was used. A drop of nanoparticle solution was placed on a standard TEM copper grid, coated with a thin film of polyvinyl formal, and dried at 296 ± 2 K for 15 min. The qualitative composition of metal nanoparticles was established by the electron diffraction patterns (Supplementary Figures S1 and S2).

Nanoparticle size distribution and their ζ-potential were analyzed by dynamic light scattering with a Zetasizer Nano ZS (Malvern Instruments, Worcestershire, UK). Measurements were realized in a 1-mL quartz cuvette or universal U-shape polystyrene cuvette at 25 °C (He-Ne laser, wavelength 633 nm) in triplicate.

To determine the mass concentration of metals in dispersed phase, they were completely ionized by concentrated HNO_3_ for 3–4 min immediately before measurement. Metal concentration was analyzed using an atomic-emission spectrometer, iCAP 6300 Radial View (Thermo Fisher Scientific Inc., Waltham, MA, USA), with inductively coupled plasma. The instrument was calibrated with standard solutions of Ag^+^ (328.07 nm), Ta^5+^ (240.06 nm), Cu^2+^ (324.75 nm), Fe^3+^ (238.20 nm), Ti^2+^ (323.45 nm), and Zn^2+^ ions (206.20 nm), and a 3 vol.% HNO_3_ was used as a background. A shift of calibration curves was checked every 15 min.

### 2.3. Fibrous Materials and Their Characteristics

The fibrous materials, according to the manufacturer, contains 30% cotton and 70% meta polyaramide fiber fabric and is covered by poly(vinylidene difluoride)-*co*-poly(tetrafluoroethylene) membrane (Teks-Centre Ltd., Moscow, Russia). The percentage of surface coverage by a membrane was determined using a scanning electron microscope, Vega3 SB (Tescan, Brno, Czech Republic).

The structure of fibrous materials before and after application of metal nanoparticles were studied using a scanning electron microscope, Vega3 SB (Tescan, Brno, Czech Republic), with an energy-dispersive analyzer, X-Act 10 mm^2^ SDD Detector (Oxford Instruments, Abingdon-on-Thames, United Kingdom). The linear profiles and two-dimensional maps of the spatial distribution of individual elements were revealed from X-ray spectrum of characteristic lines (or photon energies) of the elements present with an accuracy of about 1% and a detection limit of 0.01%. Analysis of the images was realized using a package, Fiji (a distribution of ImageJ, freely available at https://imagej.net/Fiji/Downloads).

The water vapor transmission rate through material was determined according to ISO 2528:2017.

Metal nanoparticles were deposited onto material dropwise and then dried at a room temperature within Petri dishes. To limit unnecessary losses, the maximum loading volume of the single deposition was limited to a 50 μL/cm^2^. Alternatively, material was immersed into the nanoparticle solution as a whole until steeped in, and then removed and dried.

### 2.4. Investigation of Bactericide Activity

Cytotoxicity of nanoparticles towards mammalian cells was analyzed by MTT assay [15] with a mouse fibroblast NIH/3T3 (ATCC CRL-1658). Briefly, cells were cultured and diluted using a complete growth medium (DMEM with the addition of 10 vol.% fetal bovine serum and antibiotics). The grown cells at a concentration of 2 × 10^5^ cells/mL were placed in a 96-well microplate and incubated for 24 h at 37 °C for adhesion to the surface. Further, a 150 μL of nanoparticle solution diluted to a concentration of 0.125–8.0 μg/mL was added and incubated for 24 h at 37 °C. After that, a 10 μL of MTT solution (5 mg/mL in DPBS buffer) was added to each well and incubated for 1 h. Next, the medium was gently decanted, and a 100 μL of DMSO was added to the each well. The optical density of the samples in the wells was measured on a microplate reader at 492 nm. Each experiment was carried out in triplicate; wells without metal nanoparticles were used as a control.

Biocide activity of metal nanoparticles towards luminescent bacteria were measured using the known method [16] with a *Photobacterium phosphoreum* B-1717 (All-Russian Collection of Industrial Microorganisms) immobilized in a poly(vinyl alcohol) cryogel. Briefly, the granules of immobilized bacteria were placed into a 100 μL of 0.3–30,000 ng/mL metal nanoparticle in a 2% NaCl water solution and incubated at 10 ± 1 °C for 30 min. The residual intensity of bioluminescence (*I*/*I*_0_) was analyzed with a Microluminometr 3560 (New horizons diagnostics, Arbutus, MD, USA). Control granules were treated analogously but without nanoparticle addition.

The standard disk diffusion test was applied to a *Bacillus cereus* 8035 (ATCC 10702) and *Staphylococcus aureus* subsp. aureus (ATCC 25178) onto agar medium based on the Hottinger broth. Samples of fiber materials were aseptically deposited via backside or front face onto agar immediately after its inoculation by bacteria. After that, Petri dishes were incubated for 24 h at 37 °C and the growth inhibition zone was measured. Fiber material without nanoparticles was used in the same way as a control.

To quantitatively determine the bactericide activity of metal nanoparticles, the original method was applied to cells of gram-negative bacteria *Escherichia coli* DH5α (Thermo Fisher Scientific, Waltham, MA, USA) and gram-positive bacteria *Bacillus subtilis* B-522 (All-Russian Collection of Microorganisms, Russia). Cells were cultured in Luria–Bertani (LB) growth medium on a thermostatically controlled shaker, Adolf Kuhner AG (Basel, Switzerland), at 37 °C and agitation of 150 rpm. Cell growth was monitored using a spectrophotometer, Agilent UV-8453 (Agilent Technology, Waldbronn, Germany), at 540 nm. Bacterial cells were grown for 18–20 h and then sterilely separated from the culture broth by centrifugation at 8000× *g* for 10 min (Avanti J25, Beckman, Miami, FL, USA). Cell biomass was resuspended in a sterile 0.9% NaCl to a concentration of 10^6^ cells/mL.

A 100 μL of the cell suspension was added to a 100 μL of 0–10 μg/mL of metal nanoparticles (with preliminary organic solvent evaporation) and incubated at 36 ± 2 °C for 3 h. The minimum bactericidal concentration (MBC) was assumed as the lowest concentration of metal nanoparticles, which completely inhibited the growth of bacteria (i.e., the concentration of intracellular ATP is equal to 0). Benzethonium and benzalkonium chlorides were used as reference compounds in MBC detection. The concentration of intracellular ATP was determined by the known luciferin-luciferase method [12] with a Microluminometr 3560 (New horizons diagnostics, Arbutus, MD, USA) using a standard ATP reagent based on recombinant firefly luciferase (Lumtek LLC, Moscow, Russia). The data were linearized in semi-logarithm coordinates [11]. The mean and standard deviation (±SD) were calculated with SigmaPlot (ver. 12.5, Systat Software Inc., San Jose, CA, USA) from three independent experiments.

To study the bactericide activity of fibrous materials functionalized by nanoparticles, a new original method was used. Metal nanoparticles in various amounts (concentrations, volumes, application rates) were applied to the material samples (1 cm × 1 cm) and dried at room temperature for 20 h. Then, 50 μL of a suspension (10^6^ cells/mL in a 0.9% NaCl) of *B.subtilis* B-522 or *E. coli* DH5α was applied to the materials and incubated for 24 h at room temperature. ATP was extracted by a 1 mL DMSO for 3 h and analyzed as described above. Samples without nanoparticles or with benzetonium chloride or benzalkonium chloride were used in the same way as controls. MBCs were calculated as previously described.

## 3. Results

### 3.1. Characteristics of Metal Nanoparticles and Their Deposition onto Fibrous Material

Metal nanoparticles obtained in electric arc have variable characteristics (Table 1). All of them tend to aggregate while increasing their concentration, and according to DLS measurements, they can grow up to μm size (Supplementary Table S1). Simultaneously their ζ-potential increases also.

To achieve high surface concentration of nanoparticles within fibrous material, it can be realized via different ways. Simultaneous synthesis and deposition of Cu nanoparticles was published previously [17]. However, it was inconvenient in this work due to application of preformed fibrous material. Alternatively, initial high enough concentration of nanoparticles and/or multiple deposition cycles were used to obtain necessary quantities of nanoparticles.

**Figure 1:**
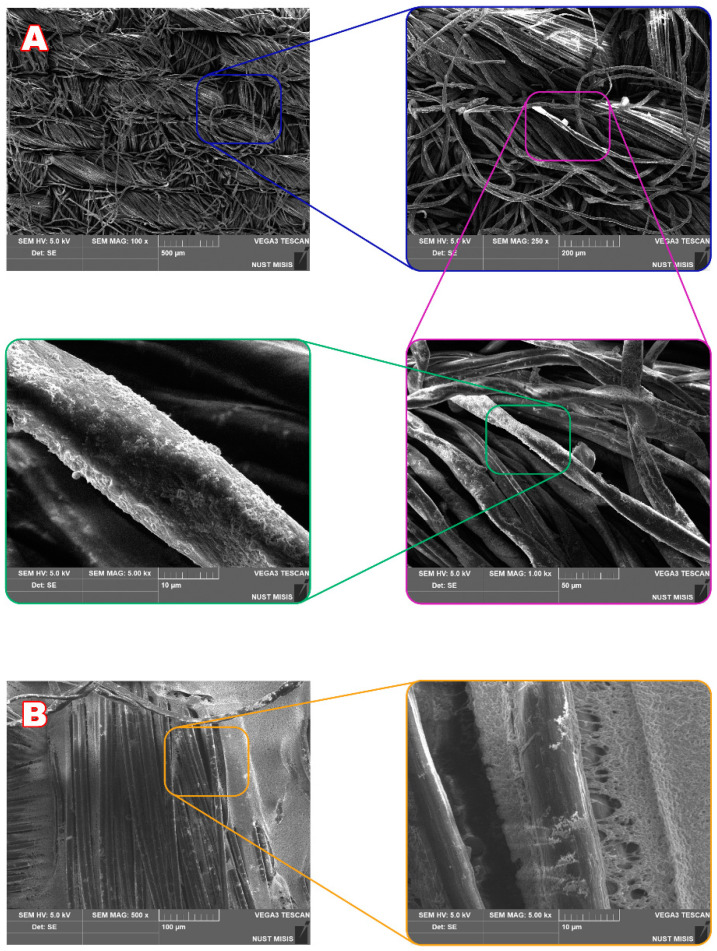
(**A**) SEM images of the front side of the fibrous material after deposition of Ta^EtOH^ nanoparticles. (**B**) SEM images of the back side of the fibrous material after deposition of Ta^EtOH^ nanoparticles. Initial material contained 30% of cotton and 70% of meta polyaramide fiber fabric and is covered by poly(vinylidene difluoride)-*co*-poly(tetrafluoroethylene) membrane from the back side.

The outer hydrophobic polymer membrane was not continuous and covers ca. 50% of the surface of the fibrous materials used (Figure 1). Moreover, it has pores of 0.45–0.65 μm which ensure high water vapor transmission rate of ca. 480 g/(m^2^ × d).

**Figure 2:**
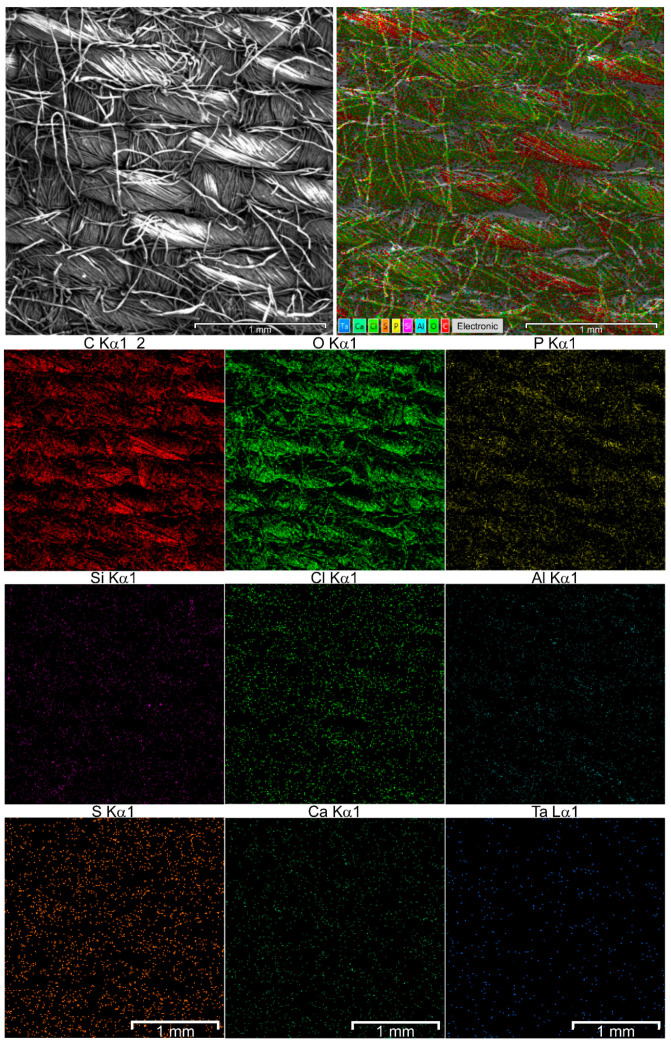
SEM and composite elemental images of fibrous material after deposition of Ta^EtOH^ nanoparticles. Elementwise chemical analysis is shown on inserts.

Nanoparticles (for example, of Ta) were regularly distributed on the surface of fibrous material (Figure 2), though Ta aggregates tend to be accumulated in close proximity with phosphate and/or carboxy anions (Supplementary Figures S3–S5). Namely, the least quantity of ‘free’ aggregates (i.e., those which were not superimposed with other elements) of Ta^EtOH^ nanoparticles was in the case of oxygen (ca. 20–30%), followed by carbon (ca. 30–40%) and phosphorus (ca. 50%). Co-localization of Ta^EtOH^ nanoparticles with other elements issued was sparser.

### 3.2. Analysis of Bactericide Activity

Initially no cytotoxicity of metal nanoparticles at concentrations of 0.125–8 μg/mL was revealed using MTT assay with mouse fibroblast NIH/3T3 cell line (Supplementary Figure S6).

**Figure 3:**
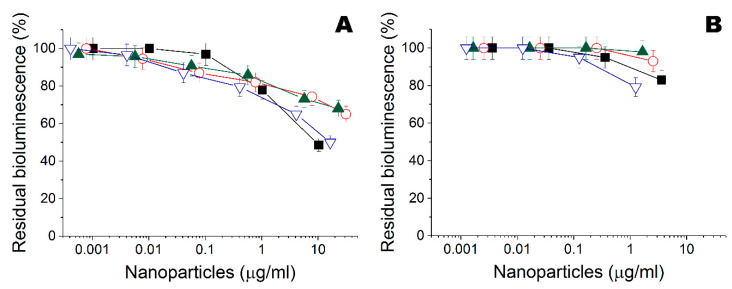
(**A**) Residual intensity of bioluminescence of immobilized cells *Photobacterium phosphoreum* treated by the Fe (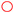), Ta (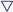), Ti (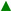), and Zn (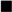) nanoparticles obtained in ethanol. (**B**) Residual intensity of bioluminescence of immobilized cells *P. phosphoreum* treated by the Fe (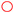), Ta (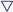), Ti (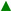), and Zn (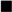) nanoparticles obtained in water.

After that, their cytotoxicity was issued with a luminescent *Photobacterium phosphoreum* immobilized within a poly(vinyl alcohol) cryogel [16,18]. Cytotoxic effect was variable, depending on the metal used (Figure 3), and was maximal for Ta and Zn nanoparticles.

**Figure 4:**
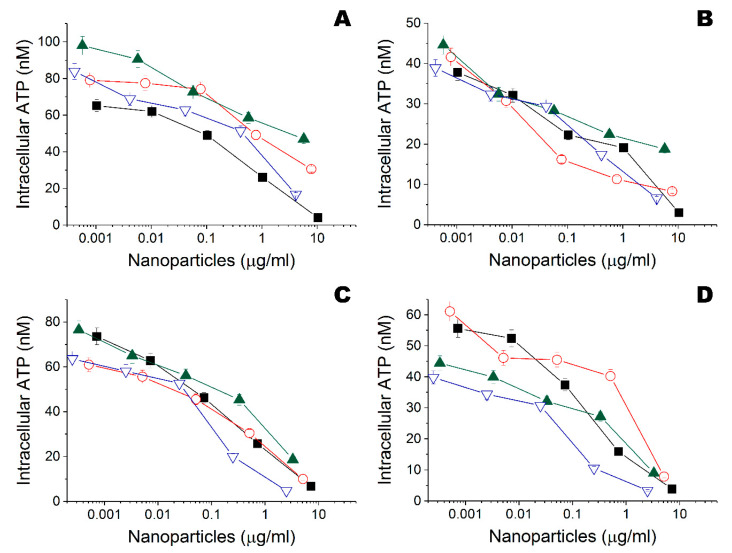
(**A**) Influence of Fe (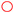), Ta (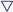), Ti (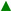), and Zn (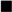) nanoparticles obtained in ethanol on intracellular ATP of *Escherichia coli* DH5α. (**B**) Influence of Fe (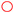), Ta (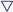), Ti (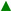), and Zn (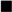) nanoparticles obtained in ethanol on intracellular ATP of *Bacillus subtilis* B-522. (**C**) Influence of Fe (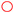), Ta (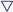), Ti (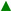), and Zn (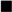) nanoparticles obtained in water on intracellular ATP of *Escherichia coli* DH5α. (**D**) Influence of Fe (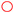), Ta (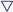), Ti (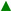), and Zn (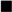) nanoparticles obtained in water on intracellular ATP of *Bacillus subtilis* B-522.

According to preliminary results with disk diffusion assay, functionalized materials had some bactericide activity towards both *Bacillus cereus* 8035 and *Staphylococcus aureus* subsp. aureus cells (Supplementary Figures S7–S10). However, this method appeared to be insufficient for the purposes of the work. Further bactericide activity of metal nanoparticles was analyzed against gram-negative (*Escherichia coli*) and gram-positive (*Bacillus subtilis*) bacteria using the original method (Figure 4). Gram-negative *E. coli* were more resistant, compared to gram-positive *B.* *subtilis*. Ta and Zn nanoparticles obtained both in ethanol and water had the best biocide potency and MBCs of 4–42 μg/mL and 10–27 μg/mL, respectively (Table 2). The difference of biocide activity between these nanoparticles obtained in ethanol or in water against gram-negative *E. coli* and gram-positive *B.* *subtilis* cells was not statistically significant: *p* = 0.100 and *p* = 0.700 in ethanol, and *p* = 0.100 and *p* = 0.200 in water according to ANOVA on ranks, respectively.

**Figure 5:**
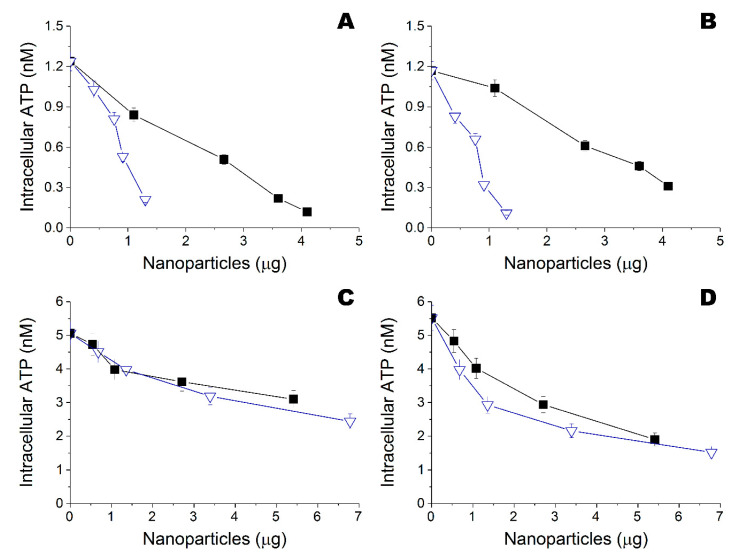
(**A**) Influence of Ta (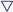) and Zn (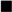) nanoparticles obtained in ethanol and deposited onto fibrous material, on intracellular ATP of *Escherichia coli* DH5α. (**B**) Influence of Ta (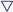) and Zn (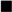) nanoparticles obtained in ethanol and deposited onto fibrous material, on intracellular ATP of *Bacillus subtilis* B-522. (**C**) Influence of Ta (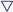) and Zn (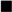) nanoparticles obtained in isopropanol and deposited onto fibrous material, on intracellular ATP of *Escherichia coli* DH5α. (**D**) Influence of Ta (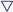) and Zn (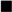) nanoparticles obtained in isopropanol and deposited onto fibrous material, on intracellular ATP of *Bacillus subtilis* B-522.

Nanoparticles retained some (12.5–20%) of their bactericide activity after deposition onto fibrous material (Figure 5; Table 3), and Ta nanoparticles have demonstrated better performance. Under the same conditions, the usual antibacterials (benzalkonium and benzethonium chlorides) had MBCs of ca. 100–150 pg/cell that were orders of magnitude worse than values in suspension 0.6–16 pg/cell [19].

## 4. Discussion

Zn, Fe, and other nanoparticles are known to inhibit bioluminescence of *P. phosphoreum* and other luminescent bacteria [20,21]. However, to the best of our knowledge, there is no such information about Ta nanoparticles. Depending on the test microorganism used, variable sensitivity could be achieved [22].

Immobilized *P. phosphoreum* were quite sensitive and could recognize the most potent biocide nanoparticles at the preliminary step already. Interestingly, bioavailability of nanoparticles for immobilized cells seemed to depend on the solvent used to obtain them, being maximal in ethanol. It could be caused by increased ζ-potential of the most nanoparticles (except Fe) obtained in water (Table 1). On the one hand, biocide activity of nanoparticles towards suspension cells was usually enhanced at large ζ-potential [23]. On the other hand, these immobilized cells were shielded by additional polymer matrix which could tightly bind such strongly charged nanoparticles. The real example of a similar mode of action is a bacterial biofilm formation, which is known to protect cells against nanoparticles toxicity [24]. Thus, nanoparticles with lower ζ-potential could be more useful for treating complicated microbial community on the surface of some materials.

Various microorganisms have a differing susceptibility to nanoparticle biocides [10], and the modulation of reactive oxygen species (ROS) and/or heavy metal ions production are acknowledged as the main mechanisms of their antimicrobial action. Though other pathways, like enhanced penetration into cell [25] or influence on cell envelope [26], etc., are also possible.

Zn, Fe, and Ti nanoparticles are extensively investigated [27,28] but only few works deal with Ta nanoparticles. Previously Ta_2_O_5_ nanoparticles of 40–60 nm showed only limited bacteriostatic effect towards *Bacillus subtilis* at a dosage of 200 pg/cell [29], and no activity was detected against *Escherichia coli*, *Pseudomonas aeruginosa*, and *Staphylococcus aureus*. The efficiency of the commercial product was somehow increased by doping of Ta to ZnO nanoparticles then. However, the mechanism of antibacterial action was dramatically changed to ROS generation that is relevant to ZnO and differed from Ta alone.

Here, differently obtained Ta nanoparticles had a much more potency while comparing both with other metals (Fe and Ti) of the current work (Table 2) and with published data [29]. Only Zn nanoparticles have comparable bactericide activity in solution. Though their activity has dropped in five to six times while being applied on the fibers (Table 3). That was not happened in the case of Ta nanoparticles obtained in ethanol, but did occur for the benzalkonium and benzethonium chlorides. This again illustrates the importance of the bioavailability consideration, and support matrix could affect positively charged active compounds even more.

Highly hydrophobic carriers are supposed to cause additional limitations for biocide activity since they will decrease diffusion of the cells to the inner layers containing these active compounds. Thus, there is a balance between hydrophilic and hydrophobic characteristics of the carrier for maximal biocide efficiency. The current combination of Ta nanoparticles with cotton/polyaramide material is likely to be close to the optimal one.

## 5. Conclusions

Thus, novel fibrous materials functionalized by metal nanoparticles and having antibacterial activity towards gram-negative and gram-positive bacteria were developed. Ta^EtOH^ nanoparticles appeared to be the best choice for the maximal bactericide activity of resulting material. Moreover, viable cells were successfully determined directly onto treated fiber samples via applied ATP assay and could be recommended for other similar researches.

## Figures and Tables

**Table 1:** Size and ζ-potential of metal nanoparticles obtained in the media of ethanol, isopropanol and water. Some TEM images are presented on Supplementary Figures S1 and S2.

Metal	Size by TEM (nm)	ζ-potential (mV)	Concentration (μg/mL)
Fe^EtOH^	2–3	1.4	7.8
Fe^water^	2–3	–7.1	2.5
Ta^EtOH^	1–3	2.7	18.3
Ta^iPrOH^	1–3	0.05	15.1
Ta^water^	2–3	68.7	5.1
Ti^EtOH^	1–3	–0.8	5.7
Ti^water^	1–2	–0.02	3.3
Zn^EtOH^	2–5	6.2	71.7
Zn^iPrOH^	4–10	1.3	53.1
Zn^water^	3–5	8.1	7.2

**Table 2:** Minimal bactericidal concentrations (MBCs) of metal nanoparticles against gram-negative (*Escherichia coli*) and gram-positive (*Bacillus subtilis*) bacteria.

Nanoparticles	MBC (μg/mL)	
	*E.* *coli*	*B.* *subtilis*
Fe^EtOH^	767 ± 139	36.6 ± 22.0
Ta^EtOH^	41.9 ± 10.9	40.4 ± 22.0
Ti^EtOH^	17,200 ± 15,200	2140 ± 1890
Zn^EtOH^	20.3 ± 9.7	27.3 ± 14.2
Fe^water^	34.4 ± 24.7	19.4 ± 5.6
Ta^water^	4.0 ± 1.1	4.8 ± 2.7
Ti^water^	66.6 ± 35.3	26.8 ± 17.1
Zn^water^	23.9 ± 11.0	10.0 ± 3.9

**Table 3:** MBCs of various antibacterials deposited onto fibrous material (1 cm^2^) against gram-negative (*Escherichia coli*) and gram-positive (*Bacillus subtilis*) bacteria.

Antibacterial Agent	Fibrous Material	MBC (pg/cell)	
		*E. coli*	*B. subtilis*
Zn^EtOH^ nanoparticles	–	20 ± 10	27 ± 14
	+	105 ± 11	161 ± 43
Ta^EtOH^ nanoparticles	–	42 ± 11	40 ± 22
	+	37 ± 4	31 ± 1
Zn^iPrOH^ nanoparticles	+	8970 ± 1290	518 ± 86
Ta^iPrOH^ nanoparticles	+	2220 ± 660	551 ± 229
Benzalkonium chloride	+	152 ± 16	133 ± 15
Benzethonium chloride	+	131 ± 13	106 ± 11

## Supplementary Materials

The following are available online at https://www.mdpi.com/2079-4991/10/9/1724/s1, Table S1: Size distribution of aggregates of metal nanoparticles obtained in the media of ethanol, isopropanol and water, Figure S1: TEM and a Laue diffraction pattern images of metal nanoparticles obtained in water, Figure S2: TEM and a Laue diffraction pattern images of metal nanoparticles obtained in ethanol, Figure S3: Overlaying of Ta^EtOH^ nanoparticles with other chemical elements on the surface of fibrous material, Figure S4: Overlaying of Ta^EtOH^ nanoparticles with other chemical elements on the surface of fibrous material, Figure S5: Overlaying of Ta^EtOH^ nanoparticles with other chemical elements on the surface of fibrous material, Figure S6: Cytotoxicity of the Fe, Ta, Ti and Zn nanoparticles obtained in water towards mouse fibroblast NIH/3T3 cells, Figure S7: Results of preliminary zone inhibition test of various fiber materials deposited by different nanoparticles towards *Bacillus cereus* 8035 (ATCC 10702), Figure S8: Results of preliminary zone inhibition test of various fiber materials deposited by different nanoparticles towards *Staphylococcus aureus* subsp. aureus (ATCC 25178), Figure S9: Growth inhibition of *Bacillus cereus* 8035 (ATCC 10702) under samples of various fiber materials deposited by different nanoparticles, Figure S10: Growth inhibition of *Staphylococcus aureus* subsp. aureus (ATCC 25178) under samples of various fiber materials deposited by different nanoparticles.
