# Supplementary material for: Metal Nanoparticles for Improving Bactericide Functionality of Usual Fibers
Source: Nanomaterials (Basel). 2020 Aug 31;10(9):1724. doi: 10.3390/nano10091724 (PMC7557869; doi:10.3390/nano10091724)
Supplement: Supplementary file 1 [file nanomaterials-10-01724-s001.pdf]

Metal nanoparticles for improving bactericide functionality of usual fibers

George Frolov[1], Ilya Lyagin[2,3], Olga Senko[2,3], Nikolay Stepanov[2,3], Ivan Pogorelsky[4], and Elena Efremenko[2,3]*

[1] National Research Technological University "MISIS", Leninsky ave. 4, Moscow 119049, Russia
[2] Faculty of Chemistry, Lomonosov Moscow State University, Lenin Hills 1/3, Moscow 119991, Russia
[3] N.M.Emanuel Institute of Biochemical Physics RAS, Kosygin str. 4, Moscow 119334, Russia
[4] 48 Central Scientific Research Institute of the Ministry of Defense of the Russian Federation, Oktyabrsky ave. 121, Kirov 610017, Russia
* Correspondence: elena_efremenko@list.ru; Tel.: +7(495)9393170; Fax: +7(495)9395417

Table S1. Size distribution of aggregates of metal nanoparticles obtained in the media of ethanol, isopropanol and water. Range of main peak and location of its maxima are shown. Each one is presented in the case of multiple peaks.

Metal	Size by DLS (nm)
Fe^{EtOH}	1000–4000; max – 2000
Fe^{water}	40–120; max – 70
Ta^{EtOH}	825–2670; max – 1500
Ta^{iPrOH}	150–1300; max – 400
Ta^{water}	150–500; max – 250
Ti^{EtOH}	35–110; max – 60
	200–800; max – 500
Ti^{water}	1–2; max – 1,5
	120–250; max – 100
	150–420; max – 200
Zn^{EtOH}	106–1105; max – 170
Zn^{iPrOH}	1000–4000; max – 1800
Zn^{water}	700–1000; max – 800
	1800–4500; max – 2800

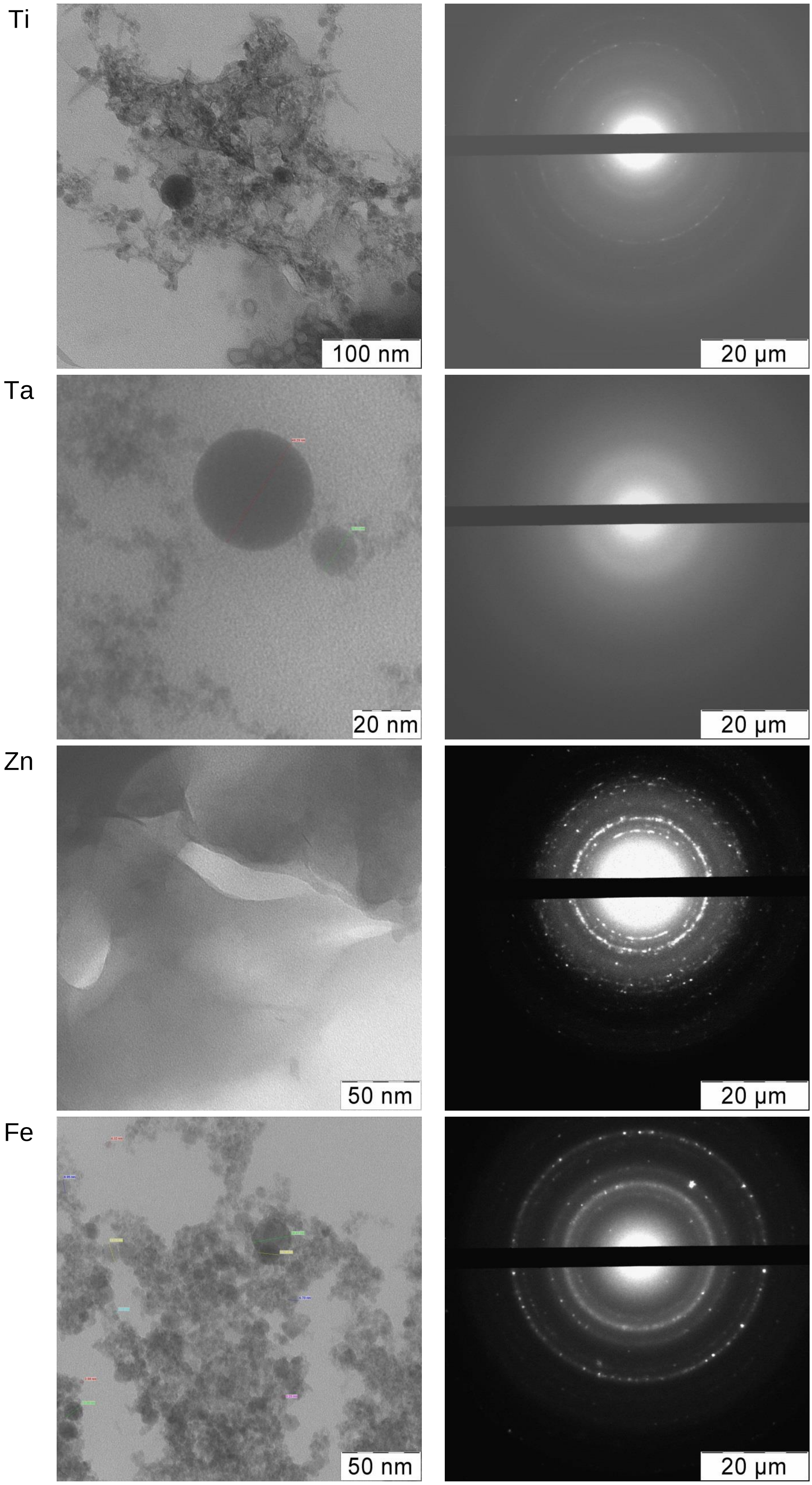

Figure S1. TEM and a Laue diffraction pattern images of metal nanoparticles obtained in water.

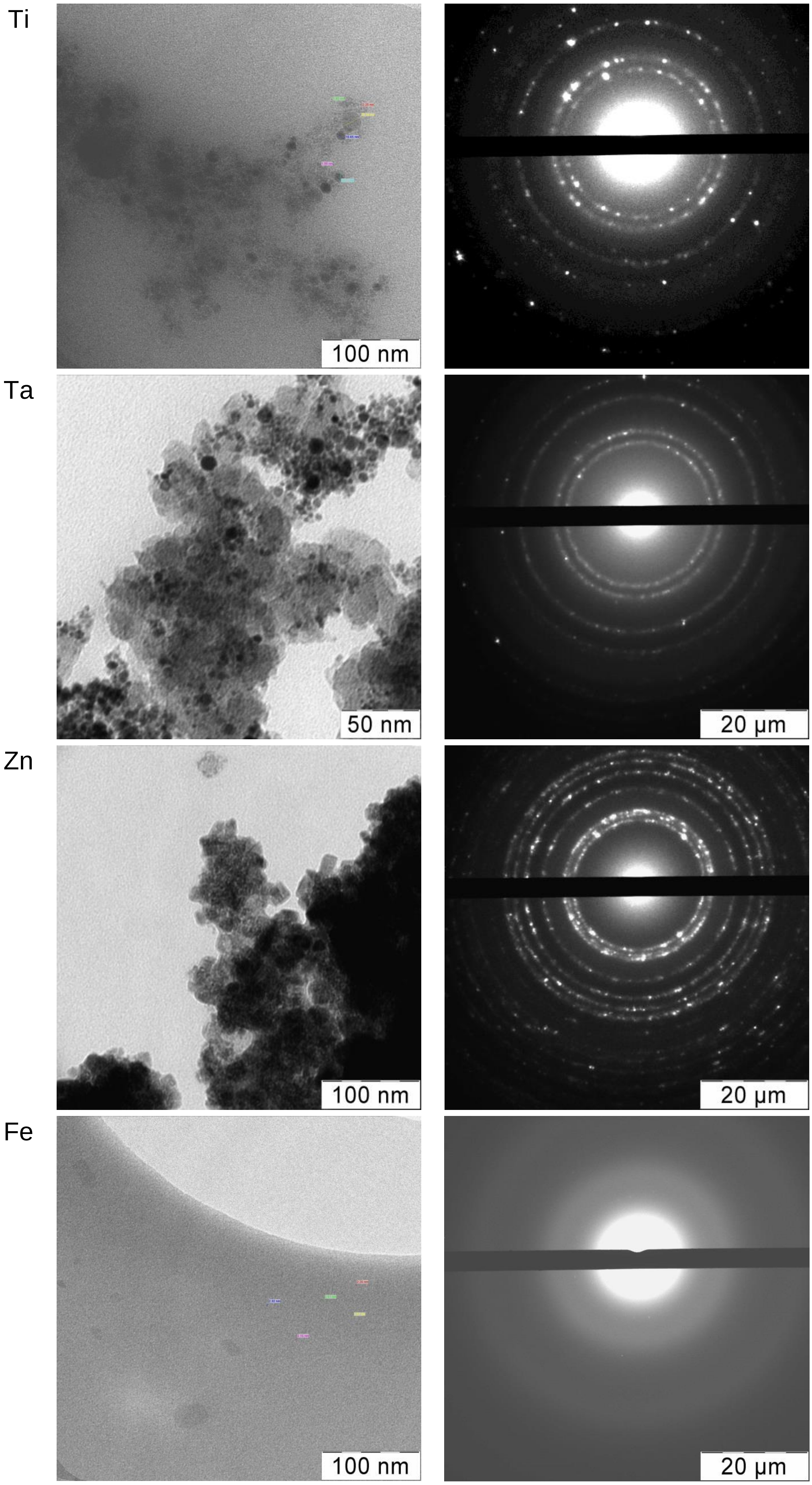

Figure S2. TEM and a Laue diffraction pattern images of metal nanoparticles obtained in ethanol.

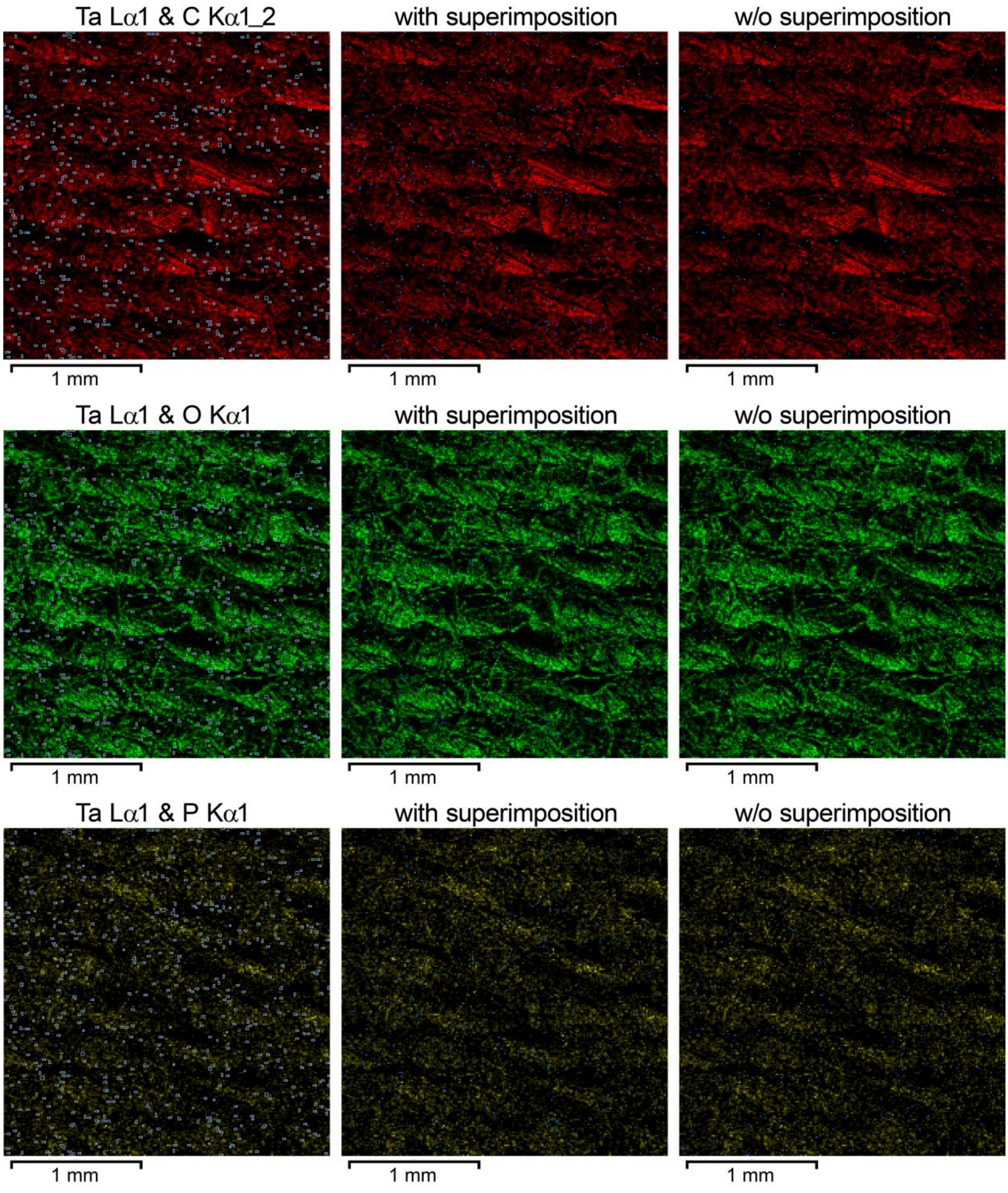

Figure S3. Overlaying of Ta^{EtOH} nanoparticles (specially highlighted for visualizing purposes) with other chemical elements on the surface of fibrous material. Individual fractions of superimposed nanoparticles are shown separately.

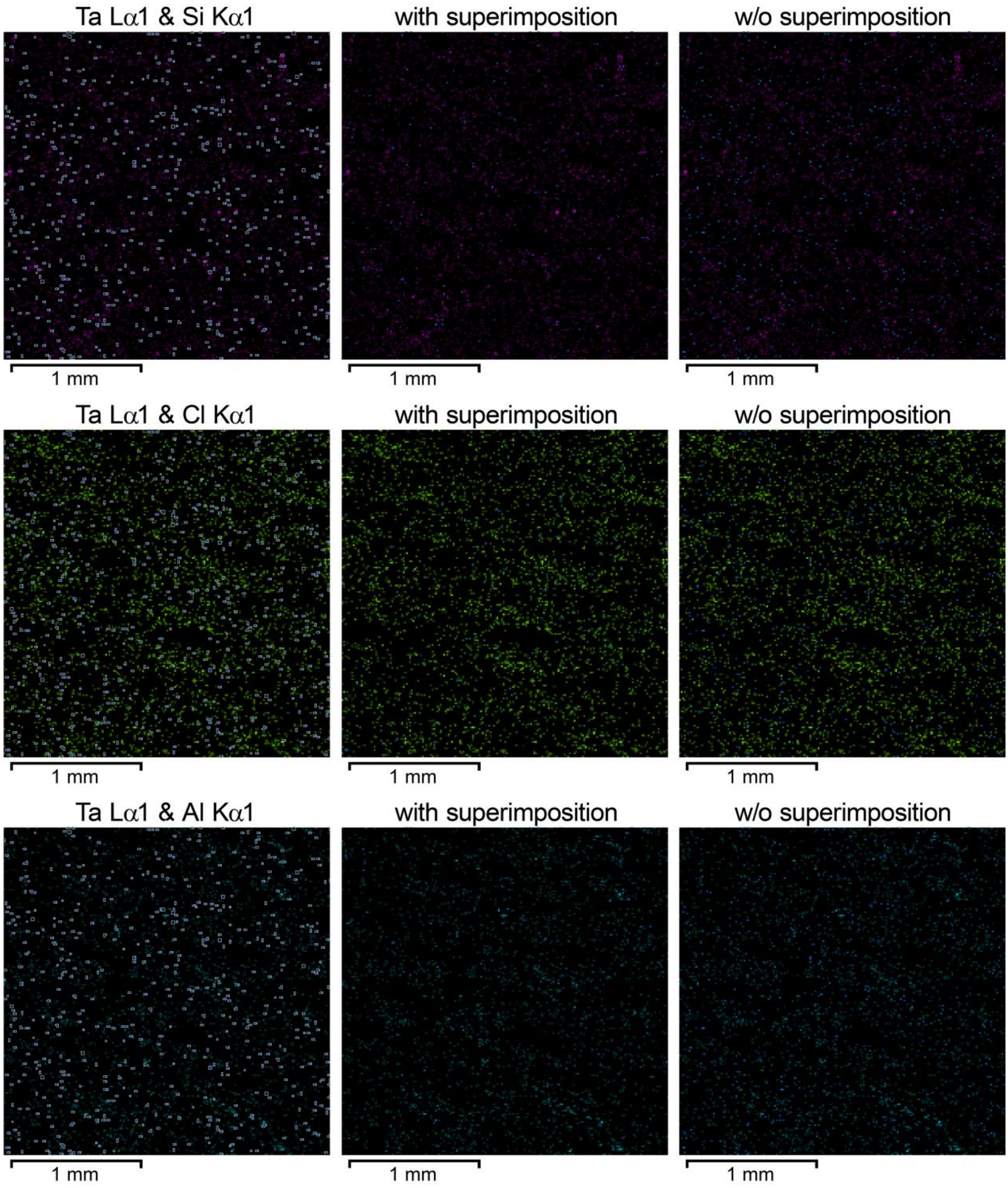

Figure S4. Overlaying of Ta^{EtOH} nanoparticles (specially highlighted for visualizing purposes) with other chemical elements on the surface of fibrous material. Individual fractions of superimposed nanoparticles are shown separately.

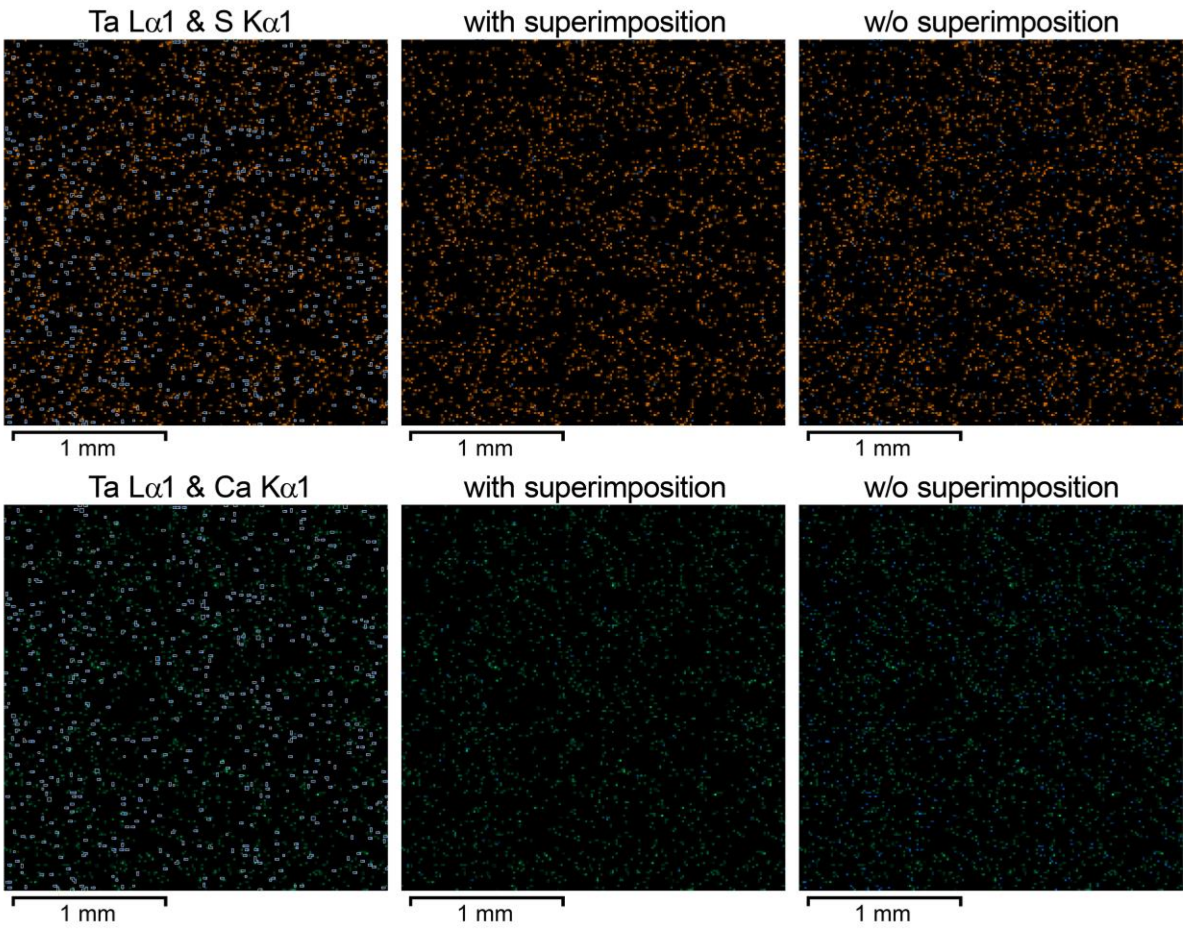

Figure S5. Overlaying of Ta^{EtOH} nanoparticles (specially highlighted for visualizing purposes) with other chemical elements on the surface of fibrous material. Individual fractions of superimposed nanoparticles are shown separately.

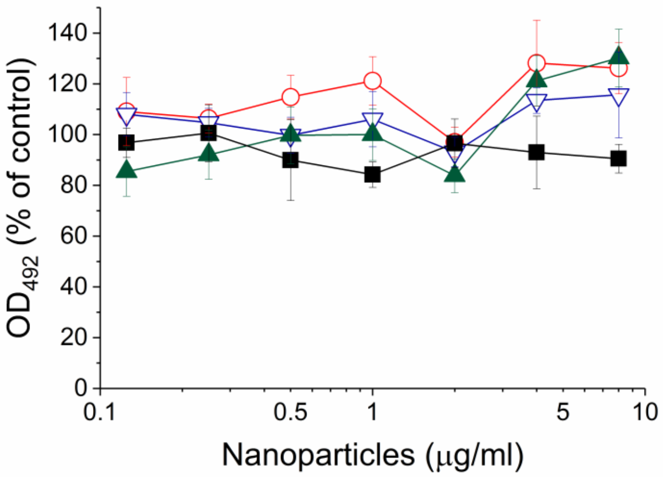

Figure S6. Cytotoxicity of the Fe (○), Ta (▽), Ti (▲) and Zn (■) nanoparticles obtained in water towards mouse fibroblast NIH/3T3 cells. Controls without nanoparticles were treated simultaneously and defined as 100%.

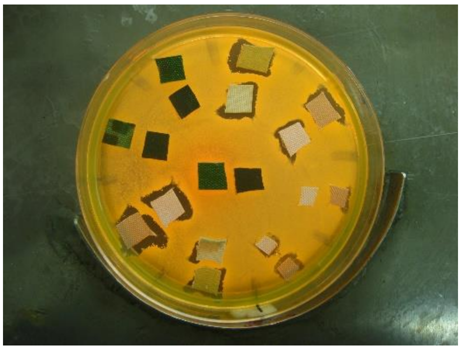

Figure S7. Results of preliminary zone inhibition test of various fiber materials deposited by different nanoparticles towards *Bacillus cereus* 8035 (ATCC 10702). Samples of fiber materials with nanoparticles were deposited via backside (outer circle) or front face (inner circle), while controls without nanoparticles were in the center.

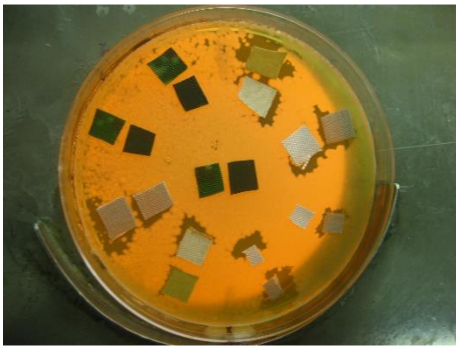

Figure S8. Results of preliminary zone inhibition test of various fiber materials deposited by different nanoparticles towards *Staphylococcus aureus* subsp. aureus (ATCC 25178). Samples of fiber materials with nanoparticles were deposited via backside (outer circle) or front face (inner circle), while controls without nanoparticles were in the center.

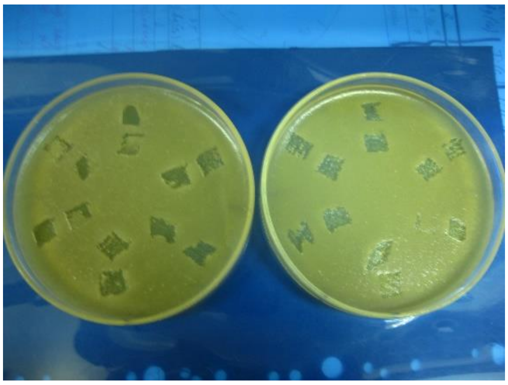

Figure S9. Growth inhibition of *Bacillus cereus* 8035 (ATCC 10702) under samples of various fiber materials deposited by different nanoparticles. Samples of fiber materials with nanoparticles were deposited via backside (outer circle) or front face (inner circle), while controls without nanoparticles were in the center.

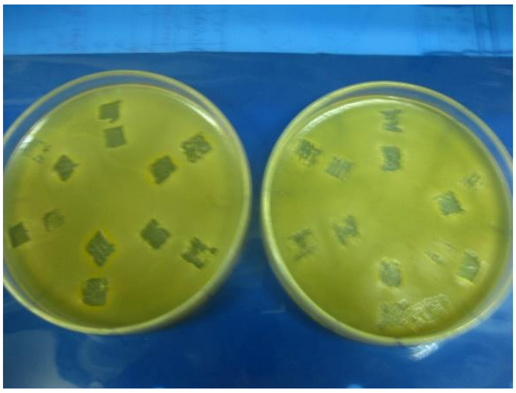

Figure S10. Growth inhibition of *Staphylococcus aureus* subsp. aureus (ATCC 25178) under samples of various fiber materials deposited by different nanoparticles. Samples of fiber materials with nanoparticles were deposited via backside (outer circle) or front face (inner circle), while controls without nanoparticles were in the center.
